# Computational Generation of Virtual Concrete Mesostructures

**DOI:** 10.3390/ma14143782

**Published:** 2021-07-06

**Authors:** Vijaya Holla, Giao Vu, Jithender J. Timothy, Fabian Diewald, Christoph Gehlen, Günther Meschke

**Affiliations:** 1 Institute for Structural Mechanics, Ruhr University Bochum, Universitätsstrasse 150, 44791 Bochum, Germany; vijaya.holla@rub.de (V.H.); thi.vu-h6d@ruhr-uni-bochum.de (G.V.); guenther.meschke@rub.de (G.M.); 2 Centre for Building Materials, Technical University of Munich, Franz-Langinger-Strasse 10, 81245 Munich, Germany; fabian.diewald@tum.de (F.D.); gehlen@tum.de (C.G.)

**Keywords:** concrete, mesoscale, modelling, virtual mesostructure, machine learning

## Abstract

Concrete is a heterogeneous material with a disordered material morphology that strongly governs the behaviour of the material. In this contribution, we present a computational tool called the Concrete Mesostructure Generator (CMG) for the generation of ultra-realistic virtual concrete morphologies for mesoscale and multiscale computational modelling and the simulation of concrete. Given an aggregate size distribution, realistic generic concrete aggregates are generated by a sequential reduction of a cuboid to generate a polyhedron with multiple faces. Thereafter, concave depressions are introduced in the polyhedron using Gaussian surfaces. The generated aggregates are assembled into the mesostructure using a hierarchic random sequential adsorption algorithm. The virtual mesostructures are first calibrated using laboratory measurements of aggregate distributions. The model is validated by comparing the elastic properties obtained from laboratory testing of concrete specimens with the elastic properties obtained using computational homogenisation of virtual concrete mesostructures. Finally, a 3D-convolutional neural network is trained to directly generate elastic properties from voxel data.

## 1. Introduction

Concrete is a highly heterogeneous composite material with a random microstructure across the length scales. This includes the material topology, size distribution, as well as their spatial configuration. Variations in the aggregate size distribution and the pore-size distribution from the nanometer scale to the decimeter scale manifest as variations in the behaviour of concrete at the macroscopic scale. The macroscopic properties of concrete such as strength, stiffness, permeability, diffusivity, etc. are completely determined by the heterogeneities in the material [1]. To enable concrete material design that is well-suited for a specific engineering application, it is important to understand and establish a clear relationship between the role of the material structure (aggregate distribution, pore-size distribution, etc.) and the macroscopic behaviour subject to various multiphysical loadings. However, establishing such a relationship using purely conventional testing methods in the laboratory is not practical due to the large variety of material compositions that have to be considered. To address this issue, several modelling and simulation approaches ranging from continuum micromechanics models [2,3,4] to mesoscale models (see for e.g., [5,6,7,8,9,10,11,12,13,14,15,16,17]), that take into account the role of the material structure in simulating the material response have been proposed. It is essential to adequately resolve the details of the material morphology at a particular scale, such that the relevant mechanisms at this scale can be correctly captured. Computational mesoscale models explicitly resolve the heterogeneity of the material and provide deeper insight into the role of the heterogeneity on the material’s behaviour especially in processes that are governed by localised phenomena, such as microcracking and failure processes. Recently, due to the advancement in computational resources and X-ray computed tomography (CT), models, in which mesostructures directly incorporated as voxel data from CT scans, have been proposed and used for numerical meso-scale simulations [18,19,20,21].

However, the process of virtually generating the mesostructure is much faster and cheaper as compared to extracting the microstructure using CT scans, provided that the virtually-generated mesostructure provides the same level of detail and information. Moreover, a method to generate realistic virtual mesostructures would significantly accelerate and support simulation models that integrate CT data into their pipelines. The aim of this paper is to present a tool to generate realistic virtual concrete mesostructures. In order to generate a virtual mesostructure, inclusions are first generated using a variety of computational methods and then assembled onto the main mesostructure volume using certain packing algorithms. Several optimised algorithms have been proposed in the last two decades for the random packing algorithm, constituting the most basic packing algorithm. Applications include integrated spherical particle kinetics models [22], molecular dynamics models [23,24], and discrete element models [25,26]. All these algorithms focus on achieving a maximum packing density for spherical inclusions. With regard to the inclusion shape (i.e., the aggregates), owing to the complexity involved in simulating realistic concrete aggregates, the inclusions are often represented by spherical or ellipsoidal shapes with random orientations embedded in a mortar matrix [8,27,28,29,30]. However, as the shape of the aggregate particle plays an important role in the local stress distribution in the mesostructure, virtual inclusions with smooth surfaces fail to capture stress concentrations due to sharp corners in concrete aggregates. To this end, attempts have been made to generate more realistic cement paste aggregates by employing dodecahedral shapes [31]. Xu et al. [22] used random polyhedrons created by extending triangular fundamentals to generate aggregates for asphalt mixture. Even though these shapes closely resemble real aggregates, when actual concrete mesostructures are studied, it is evident that these inclusions still lack important features such as multiple irregular faces and concave depressions etc. These features in concrete aggregates have been considered in [32] with regards to the aggregate shape, however restricted to a 2D representation.

In this contribution, a computational tool called Concrete Mesostructure Generator (CMG) that allows an efficient generation of ultra-realistic concrete mesostructure, is developed. A Python implementation is available here: https://pycmg.readthedocs.io/en/latest/ (accessed on 9 June 2021). The proposed methodology is calibrated and validated using a variety of data obtained from laboratory measurements of real concrete specimens. Finally, as an add-on, and one of the many possible potential applications of CMG, we develop an artificial neural network (ANN) model for directly predicting the elastic properties from voxel data of concrete mesostructures generated by CMG.

## 2. Concrete Mesostructure Generator (CMG)

The procedure for generating a virtual concrete mesostructure consists of two steps: (a) The generation of realistic concrete aggregate inclusions using irregular polyhedron geometries with concave depressions and (b) the packing of aggregates into the cementitious mortar (host material). The final mesostructure morphology is represented in terms of discrete voxels.

### 2.1. Modeling a Generic Aggregate

**Figure 1:**
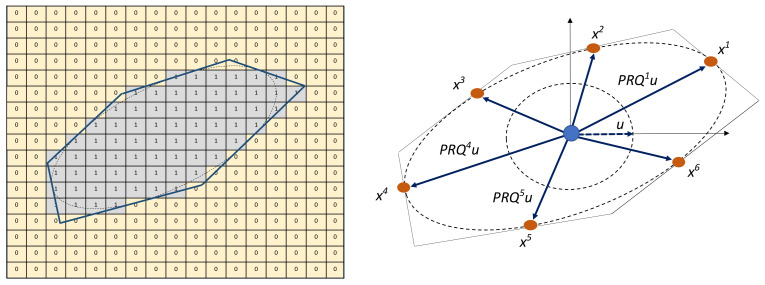
Concrete Mesostructure Generator (CMG): 2D section of a 3D polyhedron enclosing an ellipsoid in CMG (**Left**), calculation of tangent points between the ellipsoid surface and polyhedron planes with random angles (**Right**).

Coarse concrete aggregates are characterised by multiple faces, sharp corners, and irregular surfaces. Even though the shape of aggregates has a strong effect on the stress concentration, crack initiation, and propagation in concrete [33], often oversimplified models are used in most numerical analyses. In this work, we aim at modelling the aggregate topology featuring sharpness, elongation, as well as concavity by means of an irregular polyhedron geometry. The option to include an interfacial-transition-zone (ITZ) between the aggregate and the mortar matrix is also available. The procedure to generate a virtual aggregate involves the sequential reduction of an initial cuboid to a polyhedron through slicing operations tangential to an imaginary inscribed ellipsoid as shown in Figure 1 (left).

Given the maximum aggregate size $D_{max}$ in mm, we generate a 3D array of voxels of dimension l×l×l. Here, $l=D_{max}/h$ and is rounded-off to the nearest integer and its dimension is [voxels]. The parameter *h* [mm/voxel] is the resolution of the virtual mesostructure generated by the CMG. *h* is used to convert physical dimensions in SI units into voxels, its value can be set depending on the available computational resources. If *l* is an even number, then *l* is incremented by one. This is to ensure that the array contains a mid-point voxel. The voxel at the mid-point of the array at position [(l−1)/2,(l−1)/2,(l−1)/2] is set as the origin for a cartesian coordinate system in voxel coordinates.

In order to model flat and elongated aggregates, we introduce the aspect-ratio ξ. Given the aspect-ratio and the voxel dimensions *l*, we first inscribe an imaginary ellipsoid with dimensions $r_{x}=l/2,\;r_{y}=r_{x}\cdot \xi ,\;r_{z}=r_{x}\cdot \xi$. Then, the required number of faces *N* on the polyhedron representing the aggregate is specified. Subsequently, *N* points, denoted by the position vector $X^{i}$, which are located on the surface of the inscribed ellipsoid are chosen randomly (see Figure 1 (right)). The faces of the polyhedron are assumed to be tangential to the inscribed ellipsoid at these points. Each tangent plane *i* is defined using a planar equation, which is a function of the point $X^{i}$ and orientation angles $\alpha^{i}$, $\beta^{i}$, and $\gamma^{i}$. The position vectors $X^{i}$ are determined as:

(1) $$X^{i}=P\cdot R\cdot Q^{i}\cdot u,\;i=1,2,3,\ldots N,$$

with u denoting a unit vector. The rotation operator P with angles $\theta_{x}$, $\theta_{y}$, and $\theta_{z}$ determines the final orientation of the polyhedron, and the rotation operator $Q^{i}$ with angles $\gamma^{i}$, $\beta^{i}$, and $\alpha^{i}$ determines the orientation of a polyhedron face *i*. The rotation angles $\theta_{x}$, $\theta_{y}$, and $\theta_{z}$ are chosen randomly, as aggregates in concrete do not in general orient themselves along a certain axes. R is a matrix that specifies the dimensions of the ellipsoid. All geometrical operations are performed using real numbers. After having specified the final geometry of the aggregates, we round-off the real number to the nearest integer that corresponds to the discrete voxel positions. The complete expressions for the operators introduced in Equation (1) are given below:

$$u=\left[\begin{array}{c} 1\\ 0\\ 0 \end{array}\right],\;R=\left[\begin{array}{ccc} r_{x} & 0 & 0\\ 0 & r_{y} & 0\\ 0 & 0 & r_{z} \end{array}\right]\;\;with\;\;r_{x}=l/2,\;r_{y}=r_{x}\cdot \xi ,\;r_{z}=r_{x}\cdot \xi ,$$

$$P=\left[\begin{array}{ccc} cos\theta_{z} & -sin\theta_{z} & 0\\ sin\theta_{z} & cos\theta_{z} & 0\\ 0 & 0 & 1 \end{array}\right].\left[\begin{array}{ccc} cos\theta_{y} & 0 & sin\theta_{y}\\ 0 & 1 & 0\\ -sin\theta_{y} & 0 & cos\theta_{y} \end{array}\right].\left[\begin{array}{ccc} 1 & 0 & 0\\ 0 & cos\theta_{x} & -sin\theta_{x}\\ 0 & sin\theta_{x} & cos\theta_{x} \end{array}\right],\;$$

$$Q^{i}=\left[\begin{array}{ccc} cos\gamma^{i} & -sin\gamma^{i} & 0\\ sin\gamma^{i} & cos\gamma^{i} & 0\\ 0 & 0 & 1 \end{array}\right].\left[\begin{array}{ccc} cos\beta^{i} & 0 & sin\beta^{i}\\ 0 & 1 & 0\\ -sin\beta^{i} & 0 & cos\beta^{i} \end{array}\right].\left[\begin{array}{ccc} 1 & 0 & 0\\ 0 & cos\alpha^{i} & -sin\alpha^{i}\\ 0 & sin\alpha^{i} & cos\alpha^{i} \end{array}\right],\;$$

where $\theta_{x},\theta_{y},\theta_{z}\in \left[0,2\pi \right]$, and $\alpha^{i},\beta^{i},\gamma^{i}\in \left[0,2\pi \right]$.

In order to perform the imaginary cutting operation (sequential reduction of the cuboid to a polyhedron), all voxels with position vectors $X^{k}$ are set to one if they satisfy the following condition:

(2) $$\begin{array}{cc} \left[\nabla \cdot f\left(X^{i}\right)\right]\cdot \left(X^{k}-X^{i}\right) & \leq 0,\;\;i=1,2,\ldots ,N. \end{array}$$

Here, $\;f\left(X^{i}\right)=\sum {\left(X_{j}^{i}/r_{j}\right)}^{2}-1$ for j∈1,2,3 is the surface of the ellipsoid at $X^{i}$. The above expressions are evaluated at all voxel positions. The position of the *k*th voxel is denoted as $X^{k}$. (It should be noted, that $X^{i}$ is rounded to the nearest integer before performing the calculations.) Figure 1 (left) shows an illustration of a section of the 3D array after having performed the aforementioned operations.

**Figure 2:**
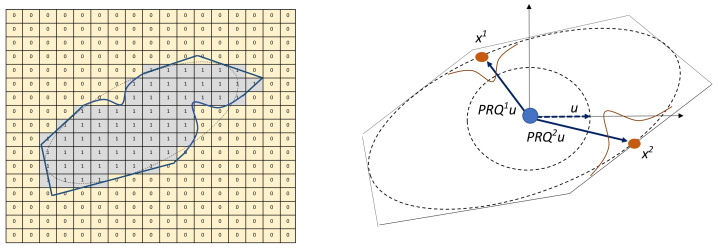
Concrete Mesostructure Generator: 2D section of a 3D polyhedron with concave depressions (**Left**), calculation of basis points on the ellipsoid surface for the Gaussian surface generation (**Right**).

Certain aggregate geometries are characterised by concave depressions, e.g., from crushing the aggregates during the production process. So far, we have designed a polyhedron whose geometry is described by voxel value = 1 embedded in a matrix material with voxel value = 0. Certain volumes of the voxel can be sculpted out to introduce concave depressions. This translates to replacing certain voxels with value 1 that are inside the volume to be sculpted out to 0. Concave depressions are introduced on the inscribed ellipsoid using the Gaussian surface equation (see Figure 2, right). The condition for setting a voxel to zero is specified as:

(3) $$\begin{array}{c} b^{i}\;exp\left(\frac{-\;(X_{2}^{g}\times X_{2}^{g}+X_{3}^{g}\times X_{3}^{g})}{\sigma^{2}w}\right)+X_{1}^{g}\times X_{1}^{g}\geq c^{i},\;for\;\;i\in 1,2,3,\ldots ,M,\\ \mathrm{where}\;\;c^{i}=X^{i},\;\;\;\;b^{i}=d\cdot X^{i},\;\;X^{g}=P\cdot Q^{i}\cdot X^{k}, \end{array}$$

where *M* denotes the total number of concave depressions centered at $X^{i}$. This equation implements a Gaussian surface below the ellipsoidal surface and checks if the voxel value is zero or not. Here $X^{i}$ are computed from Equation (1). The width of the Gaussian surface is *w*, the depth is *d*, and $\sigma^{2}$ is the variance parameter (see Table 1 for more details). Concave depressions are generated on the surface of the imaginary ellipsoid at random locations.

The procedure is as follows: First, *M* number of positions $X^{i}$ for the Gaussian surface are generated. Using these positions, the concave equations (Equation (3)) are generated with the input parameters *d* and *w*, which control the depth and width of the concavity from the ellipsoid surface. The voxel values of the points which lie inside the polyhedron and below the Gaussian surface are changed from zero to one as shown in Figure 2. $X^{i}$ is rounded to the nearest integer before performing the calculations and $X^{k}$ are the voxel positions.

A summary of all parameters for the generation of realistic generic aggregates are provided in Table 1.

In order to include the ITZ as a coating around the aggregate, the thickness *t* of the ITZ is the only input. The algorithm uses the same technique as that of the polyhedron and the Gaussian surface, but with a larger concentric imaginary ellipsoid. The thickness *t* is added to the polyhedron ellipsoid axes to obtain the coating surface,

(4) $$R=\left[\begin{array}{ccc} r_{x}+t/h & 0 & 0\\ 0 & r_{y}+t/h & 0\\ 0 & 0 & r_{z}+t/h \end{array}\right].$$

Hence, there will be two sets of concentric polyhedron and Gaussian equations. The final algorithm checks for all points in the domain first for the outer surface followed by the inner surface. Voxels at the ITZ are represented with voxel value 2. This methodology can also be used to enforce a minimum spacing between the aggregates by setting these voxel values equal to that of the matrix (host) material.

**Figure 3:**
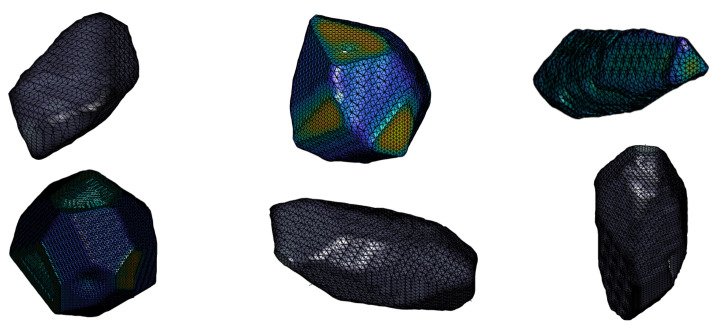
Aggregate samples generated by CMG.

**Figure 4:**
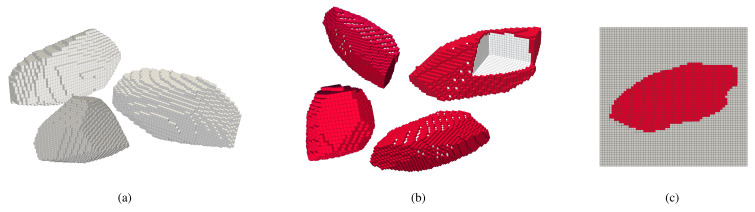
(**a**,**b**) Visualisation of the polyhedrons with maximum length of 2.5 cm (50 voxels), elongation ratio of 2.5 with 30 number of cuts, with and without coating. (**c**) Selected section featuring concave regions of the polyhedron.

Figure 3 shows aggregate samples generated by the aforementioned procedure. For a better visualisation, the surface region of the aggregates is smoothed. With multiple irregular surfaces, different aspect ratios, and concave depressions, it can be seen from these samples that the proposed algorithm can generate realistic aggregate geometries. Figure 4 shows aggregates in their original voxel form with and without a layer representing the ITZ (in red).

**Figure 5:**
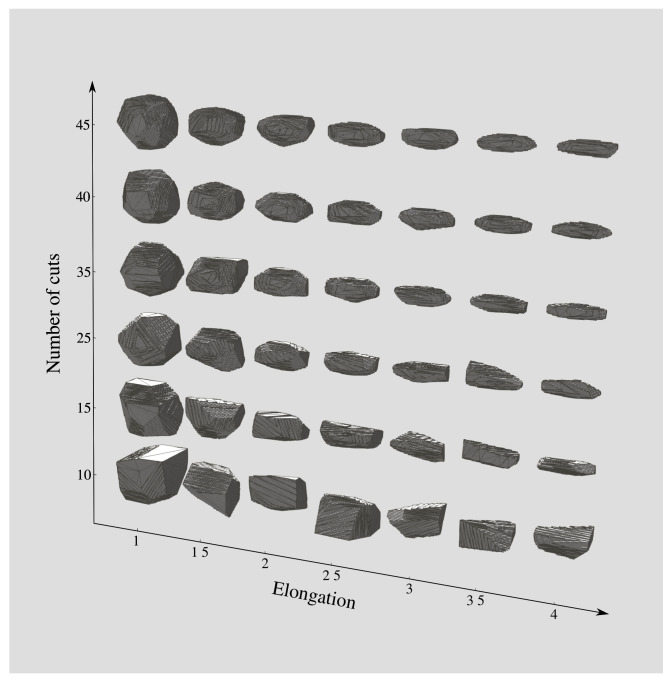
Influence of the number of cuts (*N*) and the elongation on the aggregate shape. The elongation corresponds to the value 1/ξ.

Figure 5 shows the effect of the number of polyhedron faces *N* and the elongation (1/ξ) on the aggregate shape. The aggregate size *l* of all aggregates is set to 50 voxels. The elongation varies from 1 to 4, while the number of cuts varies from 10 to 45. The aggregates are visualised using the open-source software Paraview using the decimate filter. By varying the number *N* of cuts and the aspect ratio ξ, we can obtain different types of concrete aggregates ranging from smooth-surfaced pebbles to sharp-edged aggregates that can be spherical, flat, or elongated.

### 2.2. Generating a Concrete Mesostructure

Once the aggregate is generated, it is assembled into the concrete mesostructure with a mortar matrix. The mesostructure includes both the mortar host material and aggregate inclusions in voxel format. At a given voxel position, the mortar host phase is represented with value zero and the aggregate phase with a non-zero value. The size of the voxel domain can be varied based on the requirement of the resolution and size of the mesostructure. The CMG assembly algorithm first initialises a voxel domain to the required size ($L_{x}/h$, $L_{y}/h$, $L_{z}/h$) rounded to the nearest integer filled with voxels of zeros representing the mortar host phase. Then, inclusions are generated using the aforementioned aggregate generation procedure and assembled into the domain. The basic requirements of a packing algorithm (see for e.g., [34,35]) are as follows: (a) Ensuring that the aggregates do not overlap, (b) ensuring random spatial distribution, (c) providing maximum packing density, and (d) that the mesostructure at the boundaries must be periodic.

The random sequential adsorption (RSA) algorithm [36] is used as the basis for the assembly procedure. In this algorithm, starting with the homogeneous voxelised domain, representing the mesostructure containing only the host phase, inclusions are generated and randomly placed in the domain. A random location from the 3D domain is identified and an attempt is made to include an aggregate (also in voxel format) at this location. The aggregate candidate is embedded into the current domain if there is no overlap of the aggregates. To check for overlapping, one-to-one comparison of the voxels between the inclusion matrix and that of the mesostructure matrix at the proposed assembly location is made. If all the voxels at the assembly location in the mesostructure matrix have the value of zero, then assembly is executed. According to the original RSA algorithm, if there is interference with an already assembled aggregate, a new random point is chosen and the same procedure is repeated until the particle finds a new position without any overlap with other aggregates. Once assembled, the same procedure is repeated for the newly created aggregate inclusion until the required maximum volume fraction of each inclusion size is achieved. To achieve the fastest possible assembly and higher packing density, the aggregates are assembled sequentially in a hierarchic manner according to the aggregate size from largest to smallest.

As the hosting domain is incrementally populated with aggregates, the probability of finding free space to embed a new aggregate becomes increasingly small. In the original (sub-optimal) MATLAB implementation of the model, the RSA algorithm, even though it generates statistically equivalent mesostructures by randomly distributing the aggregates in the mesostructure, achieving a packing density of more than 30%, requires a significant time for computations since the assembly procedure is completely random. Hence, to achieve a higher packing density in less computation time, the algorithm was modified from a random to a semi-random assembly algorithm (SRSA). According to SRSA, if the aggregate to be assembled does not find free space in the current random position, instead of finding a new random position, the current aggregate is incrementally translated along an orthogonal plane (either xy,xz, or yz) until the non-overlapping condition is fulfilled. SRSA was four times faster than the RSA algorithm in the Matlab implementation. However, in the current Python implementation available in [37], we observed no significant speed up as the base code was already significantly improved and optimised.

**Figure 6:**
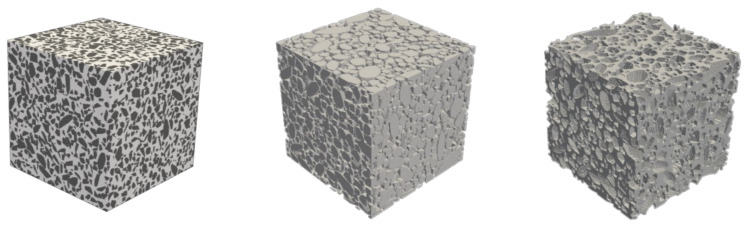
(**Left**) Virtual concrete mesostructure RVEs generated by the CMG. Visualisation of concrete mesostructures with aggregates only (**Center**) and visualisation of the mortar matrix (**Right**).

The assembly algorithm also implements periodic boundary conditions. Figure 6 shows typical concrete mesostructures showing the aggregate phase, the mortar matrix phase, and the concrete mesostructure generated by the CMG. The parameters used to generate this mesostructure are presented in the following section.

### 2.3. Data for the Calibration of the CMG

At the mesoscale, the internal material structure of concrete is characterised by a large volume fraction of aggregates (up to 60–70%) of different sizes, embedded in the cementitious matrix. One of the most conventional aggregate size distribution curves is that of standard DIN-1045 or the Fuller curves. As a result, a realistic virtual concrete sample should statistically represent such a distribution. To this end, the concrete standard AB16 (according to DIN-1045) with the largest aggregate size of 16 mm is considered. The voxel size is chosen to be 0.5 mm, i.e., the parameter corresponding to the resolutions h=0.5. Only coarse aggregates of a size larger than 3 mm onwards are explicitly resolved. The fine aggregates are embedded in the hardened cement paste to form the mortar material.

A detailed quantification of aggregate size distribution for AB16, together with the measurement of elastic properties of the investigated concrete samples was performed in the laboratory. The aggregates size distribution obtained from the measurements is summarised in Table 2. This data serves as the direct input for the packing algorithm, resulting in a total aggregate volume fraction of 48.29% that will be explicitly resolved.

The procedure for the calibration of the parameters defining the morphology of the aggregates is as follows: For each particle, the two most important parameters are the aspect ratio and the number of faces of the polyhedron *N*. As it was found that the aggregates of a larger size exhibit a wider range of aspect ratio, the aspect ratio of aggregates greater than 8 mm is randomly chosen from 1.75 to 3.25. Similarly, the aspect ratio range of aggregate sizes from 4 mm to 8 mm is within 2 to 3 and for a size of 3 mm, it is within 1 to 2. The number *N* for each size is determined by multiplying the aggregate size (in terms of voxel) by a factor whose value is either 0.5 or 0.625. It is to be noted that this range of values was chosen after performing several trials to obtain a realistic geometry. With regards to the concave depression in the aggregates, only aggregates larger than 12 mm are assumed to exhibit such a feature, with the prescribed values of 5 concave surfaces, 3 mm in width, and 2.5 mm in depth per aggregates particle. The variance parameter of the surface was chosen as $\sigma^{2}=10$. This choice of value provided the most realistic concave depressions for the considered aggregate sizes. For each successful placement of an aggregate, statistical data, such as volume fraction and number of particles of each size, are recorded as a footprint of the numerical sample. CMG can also be used as a tool for testing a certain distribution of aggregates before the actual production of concrete samples.

### 2.4. Comparison of Simulations vs. Laboratory Measurements

**Figure 7:**
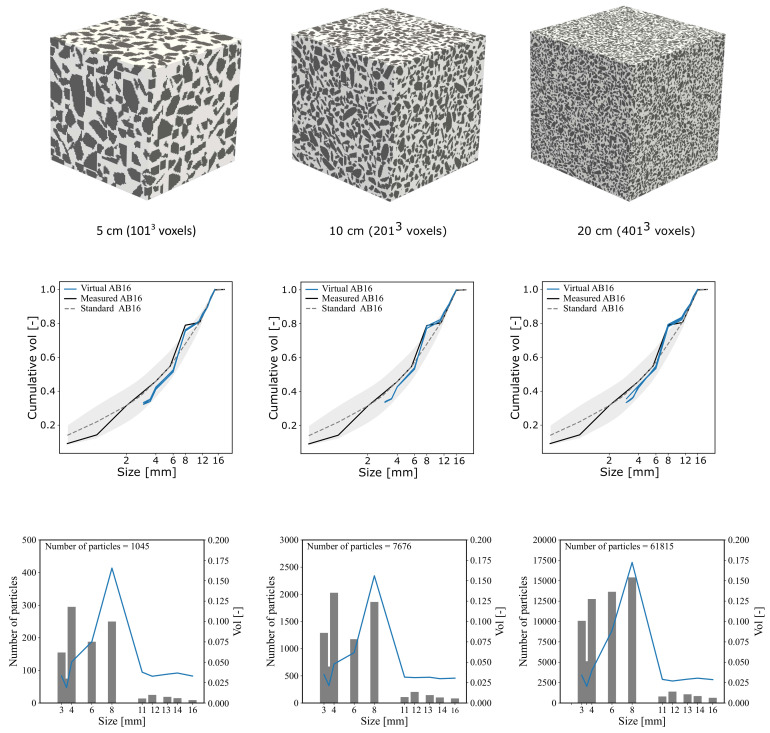
Visualisation of virtual concrete samples of size 5, 10, and 20 cm (**Top**), and their associated statistical data, namely particle size distribution curve (**Center**). Absolute volume fraction and particle counts with respect to size (**Bottom**).

In order to test the capability of the method to generate virtual AB16 mesostructures using the data from the previous section, three sample sizes of 5, 10, and 20 cm were considered. From each sample size, three specimens are generated to capture the possible stochastic fluctuation of the samples, analogous to experimental practice. The visualisation of each size is shown in Figure 7, together with the statistical information, number of particles, and volume distribution with respect to particles size. It can be seen that, in most samples, the distribution curve is in agreement with the actual grading curve obtained from laboratory measurements. The average total number of particles for each size are 1039, 7838, and 62,553 particles. The total volume fraction of these nine specimen range from 47.3% to 49.95% due to the stochastic nature of the random operations, which is acceptable, considering the computational efficiency and large number of aggregates.

**Figure 8:**
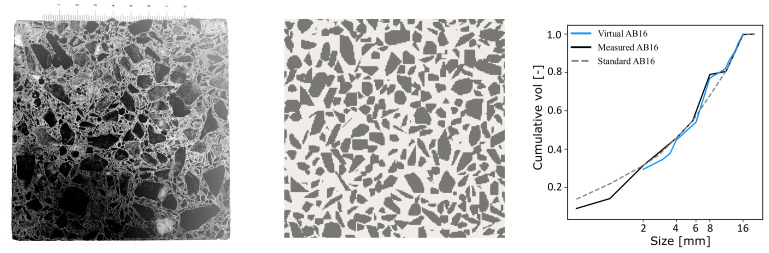
(**Left**,**Center**) Qualitative comparison of an actual concrete slice and the statistically equivalent virtual concrete generated by the CMG. (**Right**) Statistical data of the cumulative volume fraction of aggregates (laboratory measurements of AB16 vs. virtual AB16).

**Figure 9:**
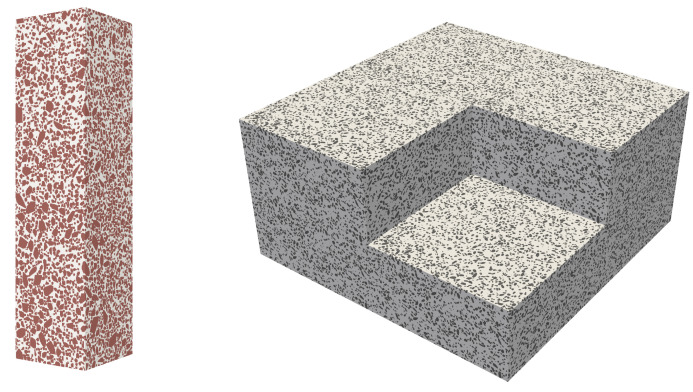
(**Left**) Virtual concrete specimens of size 10×10×40 cm discretised by 200×200×800 voxels. (**Right**) Specimen of size 60×60×30 cm, constructed by stacking eight identical periodic blocks of virtual concrete mesostructures of size 30×30×15 cm.

Figure 8 shows the actual concrete mesostructure image (Left) compared with the virtual mesostructure image (Right) generated by the CMG. Comparing the aggregate shapes, orientations, and distributions, the mesostructure image from the CMG and the actual concrete image are very similar. We can observe in both the images that the particle shapes range from an almost circular surface to highly elongated sharp-edged surfaces and that the packing density varies from less concentrated aggregate regions to densely packed regions. One can also observe concave depressions on actual concrete image similar to the CMG mesostructure. Figure 9 shows large-sized virtual concrete mesostructures according to the AB16 standard with a maximum aggregate size of 16 mm.

**Figure 10:**
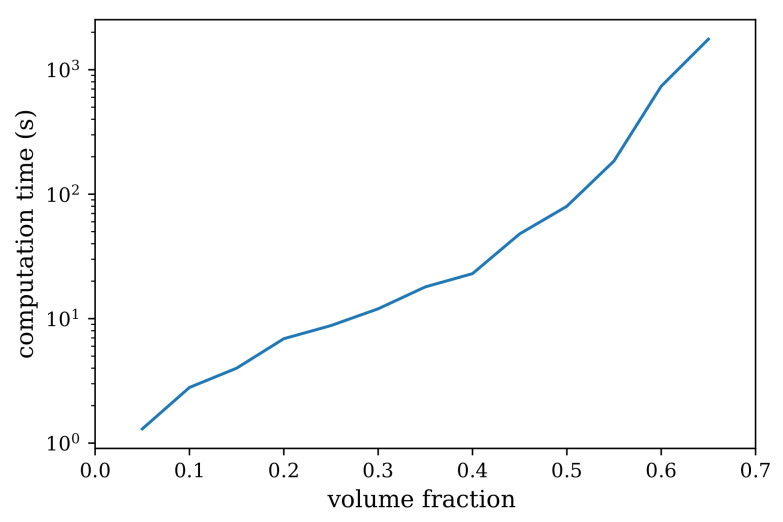
Time required to generate a virtual mesostructure using PyCMG [37] as a function of the volume fraction of aggregates on a 6-year-old Intel(R) Core(TM) i5-4210U CPU @ 1.7 GHz laptop.

The time required for generating a mesostructure of resolution 101×101×101 on a 6-year-old Intel(R) Core(TM) i5-4210U CPU @ 1.7 GHz laptop for various volume fraction of aggregates is shown in Figure 10. Computation times are hardware specific and the values shown here are expected to be the lower bound on the expected time for generating a virtual mesostructure using PyCMG [37].

## 3. Estimation of the Elastic Properties Using Computational Homogenisation

In this section we compute the elastic properties of the virtual AB16 mesostructure using computational homogenisation and validate the results with data from the laboratory. First we describe the data for model validation and then we compare this data with model predictions.

### 3.1. Data for Model Validation

In order to validate CMG, a series of tests were conducted to determine the effective properties of concrete and aggregates. The investigated concrete samples are made of Portland cement of type CEM I 52.5 R with w/c = 0.45 and one type of aggregates (Quartz) with a maximum size of 16 mm. The Young’s modulus and Poisson ratio of Quartz aggregates are measured as 84.6 GPa and 0.12. The measurement of the average Young’s modulus for the concrete samples is obtained from an uniaxial compression test. Two samples of size 10 cm were loaded using displacement control with a displacement rate 0.1 mm/h. Teflon sheets were placed between the sample and the loading platens to simulate the frictionless boundary condition. To accurately measure the true axial deformation of the sample, two external strain gauges (DD1 Displacement Transducers) were installed. The Young’s modulus was estimated using a linear regression between two points from the stress-strain curve, at 10% and 30% of the maximum compressive stress. Table 3 summarises the data obtained from laboratory measurements.

### 3.2. Computational Modeling

**Figure 11:**
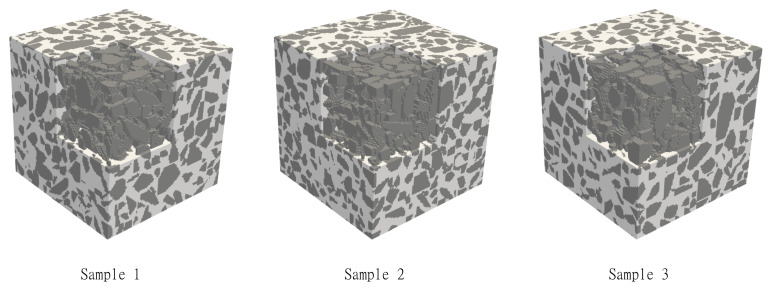
Visualisation of virtual concrete samples of size 5 cm used in the homogenisation procedure.

The elastic properties of the virtual concrete mesostructures are computed by directly using voxelised data from the CMG in a Lippmann–Schwinger-based computational homogenisation scheme (LS-FFT) [39] and a finite-cell homogenisation scheme (FCH) [40,41]. As the authors have access to both these methods, and as these methods are highly suited for voxelised data, we decided to use both these methods to compute the elastic properties of the virtual concrete mesostructure. A total of three virtual concrete samples of size 5 cm${}^{3}$ are generated (Figure 11). The aggregate volume fraction ranges from 49.66% to 47.74%, which is slightly higher than the value corresponding to laboratory measurements. This is a consequence of the preprocessing procedure in the current implementation. Before assembling the aggregate into the mesostructure, the algorithm loops over the prescribed list of aggregate sizes starting from the largest aggregate. For each size, a virtual aggregate is generated, and its volume fraction is computed. Then, the total number of aggregates in this family is estimated by dividing the total aggregate volume fraction with the volume fraction of that aggregate sample size. This value is rounded to the nearest integer as this corresponds to the number of aggregates. Thus, the total volume fraction of aggregates in the virtual concrete samples with the same prescribed grading curve would also slightly vary.

The elastic material parameters for the voxels representing the aggregate material are set to $E_{i}=84.6$ GPa and $\nu_{i}=0.12$, taken directly from the laboratory measurements. The material properties of the voxels representing the mortar host are specified as $E_{mortar}=29.67\;\mathrm{GPa}$ and $\nu_{mortar}=0.206$. These values were obtained using the Mori–Tanaka homogenisation scheme (see Appendix A for further details).

Table 4 summarises the results of model predictions and the data from laboratory measurements. The FCH homogenisation was performed using a grid size of $70^{3}$ cells with polynomial degree 1 and space-depth value 2. In the FFT-based Lippmann–Schwinger homogenisation, the virtual concrete samples of size 5 cm${}^{3}$ are discretised using 201 × 201 × 201 voxels with voxel size 0.25 mm. In all cases, the predicted $E_{hom}$ is slightly overestimated but generally still in very good agreement with the data from laboratory measurements. The Poisson’s ratio is estimated as 0.169 and 0.160 by LS-FFT and FCH respectively. Once the morphology of a certain concrete mix is fully characterised for a given experimental mixture, this comparison demonstrates that the virtual sample generated using CMG is highly realistic and representative and can provide important insight in computational simulations of microstructural damage of concrete, where the local behaviour (distributed damage around aggregates) governs the overall behaviour of the material.

## 4. Direct Computation of Elastic Properties

In this final section we explore the potential of training an artificial neural network using data generated from the numerical simulations for predicting stiffness and the poisson ratio of concrete. The aim is to directly predict the elastic properties using virtual mesostructure data i.e., directly from CMG. Machine learning (ML) techniques have successfully established linkages between the microstructure and the macroscopic property of several materials based on the data acquired from experiments and numerical simulations. Once trained, the ML model can predict the material properties at speeds comparable to that of analytical methods. Hashemi et al. [42] used artificial neural networks (ANNs) along with FEM to perform real-time homogenisation of liver tissue for simulated surgery. Yang et al. [43] developed structure-property linkages for high phase contrast composites using a convolutional neural network (CNN). Recently, Rao et al. [44] employed 3D-CNN to predict macroscopic properties of micro- and mesostructures filled with spherical inclusions. However, efforts to build structure-property relations for concrete with a realistic representation of its mesostructure are limited. To build a ML model for the direct upscaling of concrete mesostructures, a database of realistic mesostructures along with their corresponding macroscopic properties is essential. To this end, in the following section, first, the data generation process and architecture of the ML model are explained. Then, the trained model is verified by comparing the predicted values with results from high-fidelity simulations.

### 4.1. Data Generation and Pre-Processing

Assuming concrete to be an isotropic material, a 3D-convolutional neural network is trained to predict the elastic modulus ($E_{hom}$) and the Poisson ratio ($\nu_{hom}$) of concrete directly from the voxel data of the material phases. To train the model, two types of concrete standards, 9 volume fractions, 4 phase contrasts, and 20 samples for each of theses types are used. A summary of these parameters is given in Table 5. First, 360 mesostructures are generated using the CMG by varying the underlying standard, the sample count, and the volume fraction according to Table 5. These mesostructures are then homogenised for 4 phase contrasts using FCH to calculate the elastic modulus and Poisson ratio. Hence, in total, there are 360 × 4 = 1440 data points to train the model. Homogenisation is performed using a FCH grid size of 40 × 40 × 40 cells, since this size has a good trade-off between accuracy and computational time for the considered volume fractions. Using mesostructures of size 100 × 100 × 100 from CMG as inputs and 2 homogenised values from the FCH simulations as outputs, the 3D-CNN model is trained to learn structure-property relationships. However, to incorporate phase-contrast information into the input data for the 3D-CNN model, before feeding the data to the 3D-CNN model, the mesostructure from CMG is preprocessed to represent phase-contrast values by the voxel values of the mesostructure. Voxel values of the aggregates are set to a value corresponding to the phase-contrast value *p* and the value of the matrix = 1. Thus, for a mesostructure with phase contrast 4, voxel values at the aggregate positions are represented by the value 4 and voxels corresponding to the mortar phase are set to 1.

### 4.2. 3D-CNN Architecture

Training the 3D-CNN model involves choosing a set of parameters (e.g., CNN type, number of layers, filter size, stride size, activation function, loss function, etc.) depending on the application. The requirement at hand involves building a link between volumetric features of the mesostructure, such as volume fraction, shape, and distribution of aggregates, along with phase contrast information, to the elastic concrete properties.

**Figure 12:**
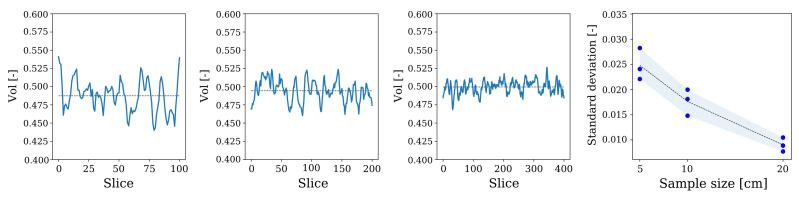
The 2D volume fraction of samples (5, 10, and 20 cm) at a different slicing position and the corresponding standard deviation of 2D volume fraction.

It can be argued that 2D slices can be used to train a 2D-CNN for generating the structure-property relationship, as this would be much cheaper. However, a 2D image analysis of virtual concrete mesostructures generated by CMG (Figure 12) shows, that the volume fraction of the mesostructure can vary up to 10% from the expected value for a mesostructure of size 100 × 100 × 100 voxels when represented as 2D images. It is observed that samples of smaller sizes exhibit higher deviation in the 2D volume fraction around its mean values, in comparison to larger-sized samples (see Figure 12). Even though a sample of 20 cm appears to have a more regular distribution throughout the volume element, a difference up to 5.2% is still encountered. Since this would deteriorate the accuracy of the CNN model and, most importantly, as this is inconsistent with the physics of the problem, a 3D-CNN model is chosen for our application.

**Figure 13:**
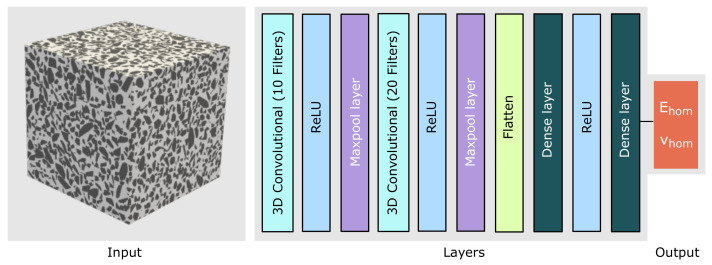
3D-CNN architecture used for training and prediction of an ML model for predicting the elastic concrete properties directly from the mesostructure of the material.

In order to choose the optimal parameters for the model, we performed a preliminary study, based on which we finally selected two 3D convolutional layers with two max-pooling layers. The architecture of the layers is given in Figure 13 and the corresponding details are summarised in Table 6. The first convolutional layer has 10 filters of size 10 × 10 × 10 and strides of size 3 × 3 × 3, the second layer has 20 filters of size 5 × 5 × 5 with strides of size 2× 2 × 2 and one maxpool layer of size 2 × 2 × 2 after each convolutional layer. Following these layers, a flattened layer and two dense layers of size 20 and 2, respectively, are considered. The final layer is of size 2 × 1. A Rectified Linear Unit (ReLU) activation function is employed for all the layers. Optimisation is performed using the stochastic gradient descent (SGD) algorithm with the mean squared error (MSE) chosen as the loss function with learning rate of 0.0005. This 3D-CNN architecture for homogenisation is implemented using Keras, a high-level neural networks API, using Python 3.7.

### 4.3. Results

**Figure 14:**
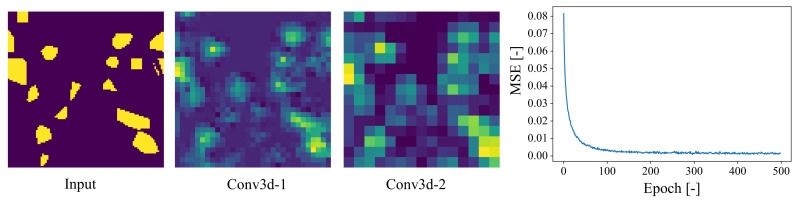
Visualisation of input slice of mesostructure with outputs from each 3D-CNN layer (**Left**); evolution of the mean squared error (MSE) vs. epochs (**Right**).

Training of the 3D-CNN model was performed on 80 Intel R Xeon R Gold 6148 CPUs. The total time for training the model was approximately 22 hours for 500 epochs with a batch size of 32. The total dataset of 1440 mesostructures with the corresponding labels (the elastic properties) is split into 1080, 180, and 180 batches for training, validation, and testing, respectively. The testing batch is used for testing the performance of the trained model since these mesostructures are completely unseen by the CNN model during training and validation. Figure 14 (left) shows how the current 3D-CNN model captures the volumetric information of the mesostructure in each CNN layer. The accuracy metric i.e., the evolution of the mean squared error (MSE) vs. epochs is plotted in Figure 14 (right). Since the errors are mean squared values, in order to achieve a high accuracy in the predictions, training was carried out up to 500 epochs. According to the graph, the CNN model has ‘learned’ the structure-property link in a mere 100 epochs with an exponential learning trend, however, comparing MSE at 100 and 500 epochs shows that the error metric value reduced by almost 50% (from 0.0034 to 0.0015), demonstrating the importance of training for higher epochs.

**Figure 15:**
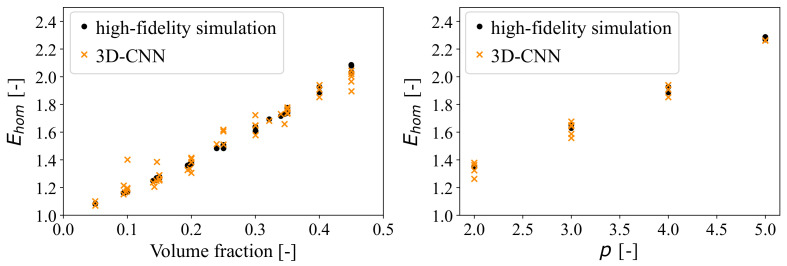
Comparison of homogenised results from FCH simulations (high-fidelity) and the ML model (3D-CNN) for various volume fractions (**Left**) and phase contrasts (**Right**).

Once the model was trained, the macroscopic properties of the new RVEs from the training set are predicted, and results are compared with actual values from FCH simulations. The mean squared error (MSE) calculated between the actual results from FCH simulations and the predicted values from the 3D-CNN are found to be 0.0048, showing an excellent prediction accuracy. It can also be seen in Figure 15 that the predicted values from the 3D-CNN are very close to the high-fidelity results obtained from FCH simulations for all volume fractions (left)) and phase contrasts (right) used in the dataset.

## 5. Conclusions

In this paper, we presented a computational tool, denoted as the Concrete Mesostructure Generator (CMG) for generating realistic virtual mesostructures for application in computational mesoscale simulations of concrete. CMG is open-source, implemented in Python, and available for all users working on mesoscale analysis of concrete structures. The results of the virtual RVE’s using the CMG have been validated by comparing the elastic properties obtained from laboratory measurements with results from two different methods of computational homogenisation (finite-cell homogenisation and Lippmann–Schwinger-based scheme) of the virtual mesostructures. An excellent agreement was obtained for both homogenisation methods. Finally, we developed a 3D-convolutional neural network model that is able to generate the elastic properties directly from virtual mesostructure images in a voxel format. The output from the trained CNN model shows an excellent agreement with results from computational homogenisation. It is concluded that the tool can be used to rapidly estimate the elastic properties of a real concrete mesostructure given either the design data from specific concrete designs, such a the grading curve, or directly using data obtained from CT scans in a voxel format. The application and use of CMG in mesoscale models that simulate distributed damage and damage identification using diffuse ultrasonic waves will be presented in a subsequent publication.

## Figures and Tables

**Table 1:** Input parameters for the CMG.

Input Symbol	Input Description	CMG Algorithm
$L_{x}$,$L_{y}$,$L_{z}$	Micro/mesostructure size in mm	Assembly algorithm
$v_{f_{max}}$	Maximum volume fraction of inclusions in micro/mesostructure	
{$l_{part}$}	Aggregate size distribution	
{$v_{f_{part}}$}	Volume fraction list	
{ξ}	Aspect ratio list	
{N}	Number of faces list	
{$S_{con}$}	Concave provision list (Yes/No)	
{$S_{ITZ}$}	Coating provision list (Yes/No)	
{d},{w}	Width and depth parameter list for the concave depression	
{t}	Coating thickness list	
$K_{max}$	Maximum number of failed assembly attempts for each particle	
*T*	Threshold to switch algorithm from RSA to SRSA	
$D_{max}$	Maximum size of aggregate	Aggregate generator—Polyhedron
*N*	Number of faces of polyhedron	
ξ	Aspect ratio of the aggregate	
$S_{con}$	Concave depression boolean (Yes/No)	
$S_{ITZ}$	ITZ provision boolean (Yes/No)	
*M*	Number of concave depressions	Aggregate generator—Concave surface
*d*	Depth parameter	
*w*	Width parameter	
$\sigma^{2}$	Variance parameter	
*t*	ITZ thickness	Aggregate generator—ITZ

**Table 2:** The composition of standard AB16 obtained from laboratory measurements.

	Cement Matrix	Fine Aggregates						Coarse Aggregates					
**Size [mm]**	**-**	**0.063**	**0.125**	**0.25**	**0.5**	**1**	**2**	**2.8**	**4**	**5.6**	**8**	**11.2**	**16**
**Volume fraction [%]**	29.259	1.504	1.619	1.758	1.758	3.634	12.174	5.0626	5.146	6.743	16.606	2.904	11.832
**Total [%]**	29.259	22.448						48.292					
**Total [%]**	**29.259**	**70.741**											

**Table 3:** Material properties of concrete and its constituents.

	Density [kg/m${}^{3}$]	Young ’s Modulus [GPa]	Poisson’s Ratio
**Cement paste ${}^{1}$**	1898	18.7	0.24
**Quartzitic aggregate**	2560	84.6	0.12
**Concrete**	2378	48.03	0.15

${}^{1}$—Material parameters of cement paste is taken from [38].

**Table 4:** Comparison of model predictions from two homogenisation methods (LS-FFT and FCH) and measured data (Lab.) for the elastic properties of concrete.

	Vol. Frac. [%]	Youngs Modulus [GPa]			Poisson’s Ratio		
		LS-FFT	FCH	Lab.	LS-FFT	FCH	Lab.
Sample 1	49.67	49.468	51.817	48.03	0.1679	0.16	0.15
Sample 2	48.73	48.983	51.331		0.1687	0.1607	
Sample 3	47.64	48.407	50.566		0.1697	0.1621	
**Average**	**48.68**	**48.952**	**51.238**	**48.03**	**0.16876**	**0.16093**	**0.15**

**Table 5:** Parameter variations for generating the dataset.

Parameters	Values
Standard	AB8, AB16
Sample count	20
Volume fraction $v_{f}$	0.05, 0.1, 0.15, 0.2, 0.25, 0.3, 0.35, 0.40, 0.45
Phase-contrast *p*	2, 3, 4, 5
total 2 × 20 × 9 × 4 = 1440 mesostructures	

**Table 6:** 3D-CNN architecture.

Layer No.	Layer Details	Input Size	Output Size
1	Conv3D, 10 filters (10${}^{3}$), strides (3${}^{3}$), ‘ReLU’	100 × 100 × 100 × 1	31 × 31 × 31 × 10
2	Maxpooling 3D (2${}^{3}$)	31 × 31 × 31 × 10	15 × 15 × 15 × 10
3	Conv3D, 20 filters (5${}^{3}$), strides (2${}^{3}$), ‘ReLU’	15 × 15 × 15 × 1	6 × 6 × 6 × 20
4	Maxpooling 3D (2${}^{3}$)	6 × 6 × 6 × 20	3 × 3 × 3 × 20
5	Flattening	3 × 3 × 3 × 20	540
6	Dense layer, ‘ReLU’	540	20
7	Dense layer	20	2

## Data Availability

Software available here: https://github.com/jtimo/pycmg (accessed on 9 June 2021).

## Abbreviations

The following abbreviations are used in this manuscript:

CMG	Concrete Mesostructure Generator
CT	Computed Tomography
ANN	Artificial Neural Network
ITZ	Interfacial-Transition Zone
RSA	Random Sequential Adsorption
SRSA	Semi-Random Sequential Adsorption
LS-FFT	Lippmann–Schwinger Fast Fourier Transform-based homogenisation
FCH	Finite Cell Homogenisation
MT	Mori–Tanaka homogenisation
3D-CNN	3 Dimensional Convolutional Neural Network
ReLU	Rectified Linear Unit
SGD	Stochastic Gradient Descent
MSE	Mean Squared Error
ML	Machine Learning

## Appendix A. Analytical Homogenisation of Mortar Matrix

As shown in Table 2, a threshold of 3 mm is set to distinguish between coarse and fine aggregates. Fine aggregates and coarse aggregate are 22.44%, and 48.292%, respectively. Subsequently, a homogenised mortar material consists of 56.59% cement matrix and 4.41% fine aggregates. Fine aggregates are generally modeled as hard spherical inclusion of stiffness $C_{i}$ and volume fraction $\phi_{i}$. According to the Mori–Tanaka homogenisation scheme, the effective stiffness tensor is computed as:

(A1) $$\begin{array}{c} C^{eff}=C_{m}-\phi_{i}C_{m}:A_{MT,i}, \end{array}$$

where $C^{eff},C_{m}$ denotes the elasticity tensor of mortar and harden cement matrix. The so-called Mori–Tanaka concentration tensor $A_{MT,i}$ of inclusions *i* can be computed with the help of the Eshelby solution S [45].

(A2) $$\begin{array}{cc} A_{MT,i} & =A_{D,i}:{\left(A_{D,i}\phi_{i}+I^{s}\phi_{m}\right)}^{-1}, \end{array}$$

(A3) $$\begin{array}{cc} A_{D,i} & ={\left(C_{m}-C_{i}\right)}^{-1}:C_{m}:{\left({\left(C_{m}-C_{i}\right)}^{-1}:C_{m}-S_{i}\right)}^{-1}. \end{array}$$

Using data given in Table 2, Young’s modulus and Poisson’s ratio of mortar with approximately 43.51% Quartz volume fraction are estimated as:

$$\begin{array}{c} E_{mortar}=29.67\;\mathrm{GPa},\;\nu_{mortar}=0.2058. \end{array}$$
